# Is Craniotomy Ever Justifiable?

**Published:** 1881-10

**Authors:** A. Reeves Jackson


CHICAGO MEDICAL

### JOURNAL & EXAMINER.

VOL. XLIII.—OCTOBER, 1881.—NO. 4.

### Original Communications.

ARTICLE I.

IS CRANIOTOMY EVER JUSTIFIABLE? By A. REEVES JACKSON, A.M., M.D., etc. Read before the Chicago Gynæcological Society, December 17, 1880.

*Mr. President and Gentlemen:*—I regret that I am obliged to commence with an apology. I have to present you with a very brief and incomplete paper,—its brevity being, however, its greatest merit. But, thinking it better to do my duty partially than not do it at all, I have brought the paper before you "scarce half made up."

I ask your attention to the consideration of the question:

IS CRANIOTOMY EVER JUSTIFIABLE?

The act of parturition in the human female must always have been one of the most interesting character; and, although we have no facts upon which to base the opinion that the process was other than physiological and safe in the very early history of our race, it seems probable that many causes must have soon come into action which were certain to occasion anxiety and danger.

Out of the perils and difficulties which came to attend parturition arose the art of midwifery. And, as we might expect, this concerned itself chiefly with the cases in which delivery could not be completed without artificial aid. When the passage of the child became obstructed nothing could be more natural than to lessen its size, and extract it by means of the hand, hooks and pincers. Accordingly, we find that the earliest obstetrical operation of which we have any mention is that of craniotomy. This operation is spoken of very fully by Hippocrates and other early writers; and, inasmuch as both turning and the use of the forceps were unknown to them, craniotomy was probably practiced in many cases where these alternatives would have been employed at a later day.

Embryotomy holds the peculiar relation to surgery that it is the only operation which is undertaken and sanctioned by the medical profession with the avowed intention of destroying a human life. All other obstetrical operations—version, forceps, Cæsarean section and other forms of laparotomy—afford a chance of safety to both mother and child. Nevertheless, this apparently cruel practice has been justified upon what seem strong and proper grounds. Even now, however, there are some conscientious practitioners who hold that the mutilation of the child is only justifiable after its death has been positively ascertained.

The common sentiment upon this point is, that when the child, living or dead, cannot be removed from the mother by the more ordinary methods,—forceps or turning,—and the dimensions of the natural pelvis are such as to permit its delivery by lessening the size of the fœtal head, such mutilation is proper and commendable; and that the life of the child, under such circumstances, should be subservient to that of the mother. This doctrine is held and promulgated, with perhaps a very few exceptions, by the leading obstetricians throughout the world.

A few months ago a somewhat remarkable paper was read before the Obstetrical Society of Dublin, by Dr. R. J. Kinkead,* in which the author takes very decided ground in opposition to these views, and maintains that, if craniotomy be ever justifiable its proper sphere is extremely limited.

* Craniotomy and its Alternatives—Cæsarean Section, Laparo-Elytrotomy, and Porro's Operation. R. J. Kinkead, A.B., M.D.—*Obs. Jl. Gr. Br. and Ireland*, March, 1880.

It is my purpose, in this paper, to notice and briefly comment upon the arguments which have been advanced by the writer referred to in support of his position. In order to do this more conveniently, I have endeavored to embody his views in a series of propositions.

Proposition 1. *The child, whilst in its mother's womb, has a separate, individual existence; has as perfect a right to its life as its mother has to hers; that our duty binds us to put its life into the scale against that of a being like ourselves, accountable to the Almighty; that, in short, we have no right to entertain the question of the relative value of one human life as compared with another.*

A fallacy underlies this argument; for, while it is true, in the abstract, that one human life is as good as another, yet, when two lives are in imminent peril and one may be saved by the sacrifice of the other,—*and in no other way*,—it seems highly proper for us to choose which shall be rescued. And this principle of action has always been recognized in the case in question; scientists and philanthropists have agreed,—generally, at least,—that the life of the mother should be held superior to that of her unborn child, and that if one must perish, it should be the latter. It is conceded that we should preferably save the strong rather than the weak; the mother, who is bound by many ties, social and moral, to her family and the world, rather than the infant without hopes, fears, affections or responsibilities. So that, while we may admit that human life should be held at the disposal of God alone, we need not forget or ignore the fact that our reason has been given us for the purpose of affording us proper guidance in cases of doubt and difficulty. And when our reason and the experience of centuries teach us that the lives of two of His creatures are mortally endangered, and that we may save one only by prompt and efficient action, we feel justified in saving that which seems to our finite judgment the most valuable.

Proposition 2. *In consequence of the teaching that the safety of the mother is always to be preferred to that of the child, resort to embryotomy has been too frequent; and attempts have been made to perforate and deliver in pelves so extremely narrow that extraction was impossible, leaving no alternative but to either*

*operate subsequently on the mother or let her die undelivered. Hence, such teaching is improper and dangerous.*

The truth of this proposition may be fully admitted—and I think it ought to be—yet it is not so much an argument against the performance of craniotomy as against its improper use. Those who have had the best teaching are not necessarily the best taught. Any surgical operation or other remedial measure may be subject to abuse, without the fact militating against the proper and judicious use of it. It is quite conceivable—quite probable, indeed—that the attempts to deliver, which are characterized by Mr. Kinkead as unwarrantable, have not been the result of carelessness but the consequence of inherent difficulties in the case. For example, a pelvis may be sufficiently large to permit the extraction of a child of usual size after mutilation; and the attempt to deliver by craniotomy would, under the circumstances, be quite justifiable, even though it might have to be abandoned subsequently owing to the fœtus being larger than common.

Proposition 3. *In the case of a woman in labor, with such a degree of pelvic obstruction as to make it evident that unless speedy aid be given, both mother and child must die, it becomes our plain duty to save one of the two so endangered if we cannot save both; hence, craniotomy being necessarily fatal should not be performed unless we are assured that, with reasonable skill in its performance, the mother's life will be spared.*

This brings us to the consideration of the question, What are the smallest pelvic diameters which will permit the extraction of a dismembered fœtus with safety to the mother? Upon this point authorities differ; for, while some state that it cannot be accomplished unless there is a clear space of three and a half by two inches, the majority fix the limit at three by two, and three by two and a half. Barnes, indeed, states that under some circumstances three by one inch may be sufficient.

It is clear that the limit should be such as would be reasonably certain to secure the safety of the mother; for, as one author urges, if craniotomy be attempted in any case in which a secondary resort to another method must be had, the danger to the mother is enormously increased; and two lives may possibly be sacrificed in the attempt to save one. But, to make choice of operation in many of these cases must be very embarrassing. It is very difficult, perhaps impossible, to ascertain within a fraction of an inch, the diameters of the pelvis, especially during labor, the time when they usually first come under notice. Then there are the considerable differences which may exist in the size of the fœtal head,—quite as important an element in the calculation, and one which we can only estimate. According to statistics collected by the late Dr. Parry, of Philadelphia, out of seventy cases of craniotomy where the smallest diameter was two and a half inches or under, the maternal mortality was about one in two and a half, nearly equal to the usual mortality from Cæsarean section.

In comparing the relative mortality of craniotomy and Cæsarean section,—the first being usually placed at one in five, and the latter at one in two and a half,—Mr. Kinkead lays great stress upon the fact that where craniotomy has been performed after long duration of labor—say forty-eight hours—the maternal mortality becomes greatly increased; and that where vain attempts to deliver have been made with forceps, it reaches a a mortality of one in two and a half, thus becoming as fatal as Cæsarean section. He proceeds, further, to show that while the tables relating to Cæsarean section show a mortality of about 57.6 per cent. they include cases in which the patients had been long in labor, some actually dying; others suffering from dangerous and deadly diseases; some in whom craniotomy had been tried and failed; and that, hence, a fair opinion as to the rate of mortality which would obtain under the best and most favorable circumstances was not at present attainable. He states, however, that in the few cases in which timely operations were performed the maternal mortality was reduced to 25 per cent. But he does not seem to consider that the tables upon which the mortality of craniotomy cases is based also include unfavorable cases,—cases in which labor has been of long duration, where the patient has been suffering from previous disease, and where unavailing attempts have been made to deliver by forceps, etc., and that if these were all included the operation would make relatively as favorably a showing compared with Cæsarean section as it does now.

Proposition 4. *In carcinoma of the uterus or vagina, a disease which must in a short time put an end to the existence of the mother, we are justified in saving the child, even though it be at the expense of some additional risk to the mother.*

Here the author makes a really strong point, and I coincide with him. The same principle which impels us to save the mother rather than the child under ordinary circumstances, should here urge us to reverse our action. We should save the best. Here, the child, if saved, would probably become a useful member of society, while the days of the mother are numbered. Embryotomy certainly destroys the child, and in this case does not save the mother; at best it could only for a brief time prolong a painful existence; and it has been shown by statistics that where craniotomy has been performed under these circumstances, it has been almost as dangerous to the mother as Cæsarean section. In reply to the argument, that because the mother's chance for living is small we have no right to still further lessen it, Mr. Kinkead aptly replies that this would be true if the child had no right to live.

Proposition 5. *When labor is complicated by the presence of uterine, ovarian or other pelvic tumors of considerable size, which are incapable of being removed or emptied of their contents, such dangerous pressure must be made upon them, even after the fœtal head has been reduced in size, that craniotomy should not be resorted to in preference to some of its alternatives—Cæsarean section, gastro-elytrotomy or Porro's operation.*

Here, too, I agree with Mr. Kinkead. Usually, in these cases, unlike those in which the deformity is bony, all the pelvic diameters are affected, and there is very great difficulty in extracting even the dismembered child. According to Stadfelt, the maternal mortality in such cases reaches 60 per cent., and when we consider that all the children are killed, the position taken by the author seems impregnable.

DR. JACKSON: In closing the discussion, I have but little to add to what has already been said. I want, however, to sincerely thank the members of the society for the very kind manner in which they have received and commented upon the paper in its present crude and unfinished form. I also thank them for the courteous permission they have given me to revise and complete it at a subsequent time.

It seems well, in these days of changing views and practice, to occasionally come back to the consideration of old landmarks. In doing so we shall find that every change proposed and practiced is not necessarily an improvement. It was because I regarded the views of Dr. Kinkead, upon the subject of craniotomy, as revolutionary and unsound, that I thought it expedient to bring them before this society for the consideration of the latter; and I am pleased to find such a unanimous expression of opinion in favor of the old views and old and tried methods. To what extent the heterodox views of Dr. Kinkead have found sympathy and support in his own country I cannot say; but, I feel like congratulating this society on the fact that they have found no encouragement here.

---

